# MiRNA-Based Regulation of Hemostatic Factors through Hepatic Nuclear Factor-4 Alpha

**DOI:** 10.1371/journal.pone.0154751

**Published:** 2016-05-02

**Authors:** Salam Salloum-Asfar, Ana B. Arroyo, Raúl Teruel-Montoya, Nuria García-Barberá, Vanessa Roldán, Vicente Vicente, Constantino Martínez, Rocío González-Conejero

**Affiliations:** Centro Regional de Hemodonación, IMIB-Arrixaca, University of Murcia, Murcia, Spain; Montana State University, UNITED STATES

## Abstract

MiRNAs have been reported as CIS-acting elements of several hemostatic factors, however, their mechanism as TRANS-acting elements mediated by a transcription factor is little known and could have important effects. HNF4α has a direct and important role in the regulation of multiple hepatic coagulation genes. Previous *in vitro* studies have demonstrated that miR-24-3p and miR-34a-5p regulate *HNF4A* expression. Here we aimed to investigate the molecular mechanisms of miR-24 and miR-34a on coagulation through *HNF4A*. Transfections with miR-24 and miR-34a in HepG2 cells decreased not only *HNF4A* but also *F10*, *F12*, *SERPINC1*, *PROS1*, *PROC*, and *PROZ* transcripts levels. Positive and significant correlations were observed between levels of *HNF4A* and several hemostatic factors (*F5*, *F8*, *F9*, *F11*, *F12*, *SERPINC1*, *PROC*, and *PROS1*) in human liver samples (N = 104). However, miR-24 and miR-34a levels of the low (10^th^) and high (90^th^) percentiles of those liver samples were inversely correlated with *HNF4A* and almost all hemostatic factors expression levels. These outcomes suggest that miR-24 and miR-34a might be two indirect elements of regulation of several hemostatic factors. Additionally, variations in miRNA expression profiles could justify, at least in part, changes in *HNF4A* expression levels and its downstream targets of coagulation.

## Introduction

High levels of coagulation factors may disturb the fragile balance of the hemostatic system leading to thrombotic events. However, coagulation factors have a substantial interindividual variability in healthy human plasma [1,2] so that the threshold for the individual thrombotic risk will come defined by the joint action of genetic, environmental, and acquired factors [1,3–5]. Among the genetic elements that drive the synthesis of coagulation factors a hereditary component has been described for several of them although the heritable basis for high or low levels of factors remains unknown [1]. Interestingly, common regulatory genes coordinate simultaneously the expression of several clotting factors which would allow to categorize individuals in those with high or low levels of coagulation factors [2,4].

A good candidate among these common regulatory genes is Hepatocyte Nuclear Factor 4α (HNF4α, NR2A1, gene symbol *HNF4A*), a member of the nuclear receptor superfamily, essential for liver homeostasis and linked to several diseases including diabetes, hemophilia, atherosclerosis and hepatitis [6–8]. HNF4α has been linked to the expression of a large number of coagulation genes such as prothrombin [9], Factor (F) VII [10,11], FVIII [12], FIX [13,14], FX [15], FXI [16], FXII [17], protein S [18], protein Z [19], and antithrombin [20,21]. The full-body *Hnf4a* knock-out mouse is embryonic lethal [22] and gene targeting using short interfering RNA (si*HNF4A*) confirmed the impact of HNF4α in regulating hepatic coagulation factors transcription [23,24].

Several inductors and repressors, that weave a complex regulatory net, participate in the expression of *HNF4A* gene [25,26]. In addition, *HNF4A* is post-transcriptionally regulated by miRNA [27,28], small non-coding RNAs implicated in protein regulation in a large number of physiological and pathological processes [29]. Takagi *et al*. were the first describing *in vitro* the regulation of HNF4α by miR-24 and miR-34a [30].

The more intuitive and better described mechanism of action of miRNAs is as CIS-suppressive-regulatory elements. Thus, up to seven coagulation factor genes have been described to be targets of miRNAs [31]. However, the hypothesis that miRNAs repress common transcription factors then working as TRANS-regulatory elements for some genes remains to be explored. Here, we aimed to thoroughly gain a deeper insight into the physiological modulator role of miRNA in the expression of downstream coagulation targets of HNF4α.

## Materials and Methods

### Cell culture and tissue samples

HepG2 cells (American Type Culture Collection, Manassas, VA) were cultured in modified Eagle's medium (MEM) (ThermoFisher Scientific, Madrid, Spain) at 37°C under 5% CO_2_. Medium was supplemented with 0.1 mM non-essential amino acids and with 10% fetal calf serum (ThermoFisher Scientific, Madrid, Spain). 104 liver samples from white donors were provided by the Research Center of Experimental Pathology Department of La Fe Hospital and CIBERehd (Valencia, Spain) and by St. Jude Children’s Research Hospital Liver Resource (Liver Tissue Procurement and Distribution System (NIH Contract #N01-DK-9-2310) and the Cooperative Human Tissue Network) [32]. None of the donors were from a vulnerable population and all donors provided written informed consent that was freely given. Human liver studies were approved by Ethics Committee for Clinical Investigation from Morales Meseguer Hospital in Murcia, Spain (#ESTU-19/12) and performed in accord with the declaration of Helsinki.

### Transfection of miRNAs

Briefly, HepG2 cells were seeded twenty four hours before transfection in complete MEM medium supplemented with 10% fetal bovine serum at 37°C in a humidified incubator with 5% CO_2_ without antibiotics and transfected with 100 nM of chemically modified double-stranded RNAs that mimic endogenous miRNAs or SCR (Life Technologies, Madrid, Spain) using PepMute transfection reagent from SignaGen laboratories (Rockville, MD). After 48 h, cells were collected for subsequent mRNA and protein analyses.

### RNA isolation and real-time RT-PCR

Total RNA was isolated using RNAzol®RT Reagent (Molecular Research Center, Inc. Cincinnati, OH). The RNA concentration and 260/280 ratio were determined by using NanoDrop spectrophotometer (Thermo Scientific, Wilmington, DE). Retrotranscription reactions were performed using 100 ng of total RNA for each sample according to the manufacturer instructions (Life Technologies, Madrid, Spain).

Quantitative real-time PCR, using TaqMan® Gene Expression Assays (Applied Biosystems, Madrid, Spain) and gene-specific primers/probes (S1 Table), was performed on a LightCycler® 480 Real-Time PCR System (Roche Applied Science, Barcelona, Spain). *F11* and *F9* were previously quantified in our previous study [33] and their mRNA expression data were used for analyses in this study. Data were analyzed using the comparative threshold cycle method (2^-ΔCt^ method) with β-actin as an endogenous reference control for quantification and normalization. To quantify expression levels of miRNAs, commercial RT-PCR assays for miR-24, miR-34a, and U6 snRNA (endogenous control) from Life Technologies were used.

### Total protein extraction and western blotting

Transfected HepG2 cells were washed twice with phosphate-buffered saline (PBS). They were then lysed in RIPA buffer [150 mMNaCl, 1 mMethylenediaminetetraacetic acid, 1% Nonidet P-40, 1% sodium deoxycholate, 0.1% sodium dodecyl sulfate (SDS), 20 mM 3-(*N*-morpholino) propanesulfonic acid (MOPS), 1 mMphenylmethylsulfonyl fluoride, pH 7.0] on ice for 20 min. The lysates were centrifuged at 12,000×g for 5 min at 4°C and the supernatants were collected for analysis of the protein concentration using a Bicinchoninic Acid (BCA) Protein Assay (Sigma-Aldrich, Madrid, Spain). These lysates (each 20 μg) were blotted and immunostained with different monoclonal antibodies: anti-HNF4α (ab92378; Abcam, Madrid, Spain) and anti-GAPDH (ab128915; Abcam, Madrid, Spain). HNF4α and GAPDH were immunodetected with the appropriate secondary antibody labeled with peroxidase (GE Healthcare, Barcelona, Spain). Western blotting detection, their corresponding densitometric analysis, and the expression of data were performed in a manner similar to that previously described [33].

### Statistical analysis

Statistical differences between groups were calculated by non-parametric Mann-Whitney U test using GraphPad Prism 6 software (GraphPad Software Inc., San Diego, CA). A p-value <0.05 was considered to be statistically significant. Correlations were observed with Pearson's correlation of coefficient and analyses were carried out using Statistical Package for Social Science (version 21.0; SPSS, Chicago, IL).

## Results

### *HNF4A* correlates with coagulation factor expression levels in human liver

**Fig 1:**
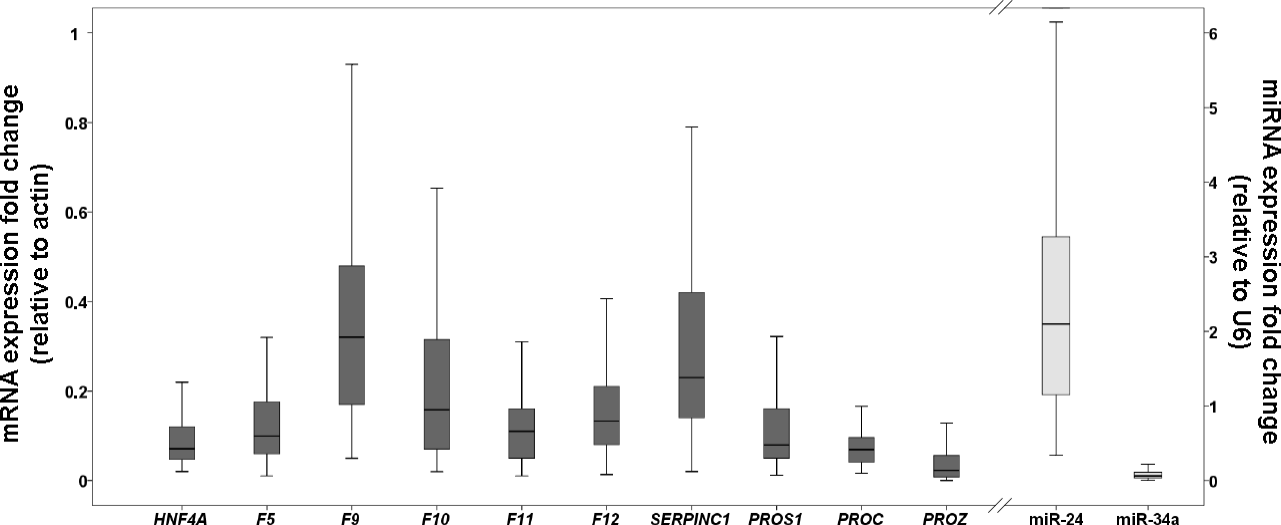
Hepatic expression profiles of mRNAs and miRNAs in human liver samples. Box plots indicate first and third quartiles of expression; the bold line in the box represents the median value; the whiskers represent the range.

To verify whether *HNF4A* modulates the expression of coagulation factors in human liver, we quantified mRNA levels of *HNF4A* and 9 genes involved in coagulation (*F5*, *F9*, *F10*, *F11*, *F12*, *SERPINC1*, *PROC*, *PROZ*, and *PROS1)* in 104 human liver samples. We observed a widespread interindividual variation in expression levels of the analyzed hepatic transcripts (Fig 1).

**Fig 2:**
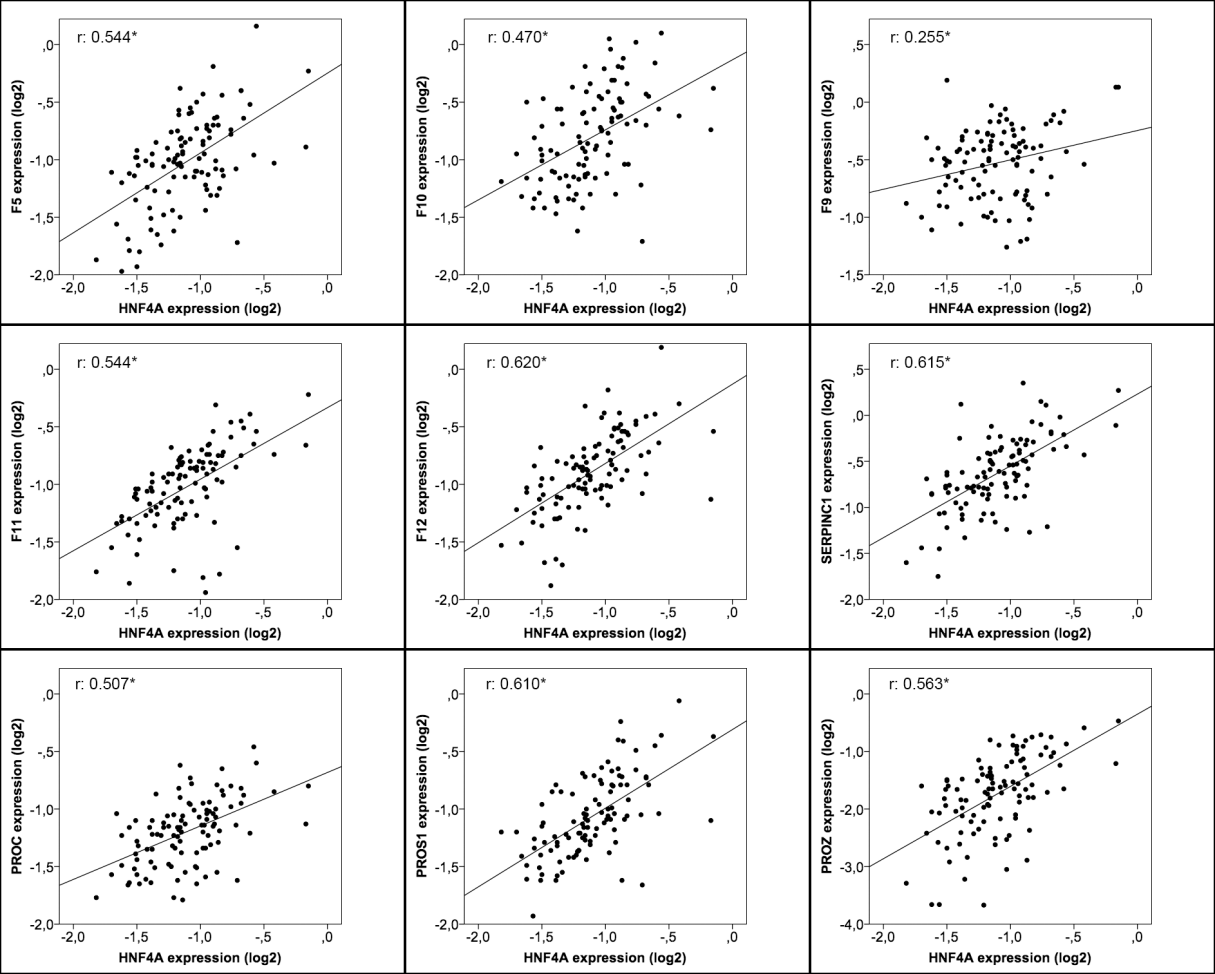
Correlation of mRNA expression levels of pro- and anti-coagulant factors with hepatic transcription factor *HNF4A*. Positive and significant correlations between procoagulant (*F5*, *F9*, *F10*, *F11*, *and F12*), anticoagulant (*SERPINC1*, *PROC*, *PROS1*, and *PROZ*) factors and *HNF4A* mRNA expression. qRT-PCR was performed in total RNA purified from healthy livers (n = 104). Each data point represents an individual liver tissue sample, and a correlation coefficient (r) is shown. The results are presented as Log2 fold change with respect to the normalization standard. The asterisk indicates a statistically significant difference (p < 0.0001).

As shown, the wider expression variability was found for *F9* (14–400%) and *SERPINC1* (6–640%) whereas *PROC* and *PROZ* had the lower range of variability (Fig 1). The distribution by percentiles according with the expression of the analyzed factors showed up to 3-fold differences among percentile 25^th^ and 75^th^ (Fig 1). Similarly, a wide variability was also observed for miR-24, as shown in Fig 1. Next, we analyzed the correlation between *HNF4A* expression and the same 9 coagulation factors in human livers. We found that those samples with higher coagulation factor expressions had also higher *HNF4A* expression (all p<0.0001; Fig 2).

These results suggest that differences in *HNF4A* expression might explain, at least in part, interindividual variations seen in the expression of coagulation factors in human liver.

### *In vitro* study in HepG2 cells

**Fig 3:**
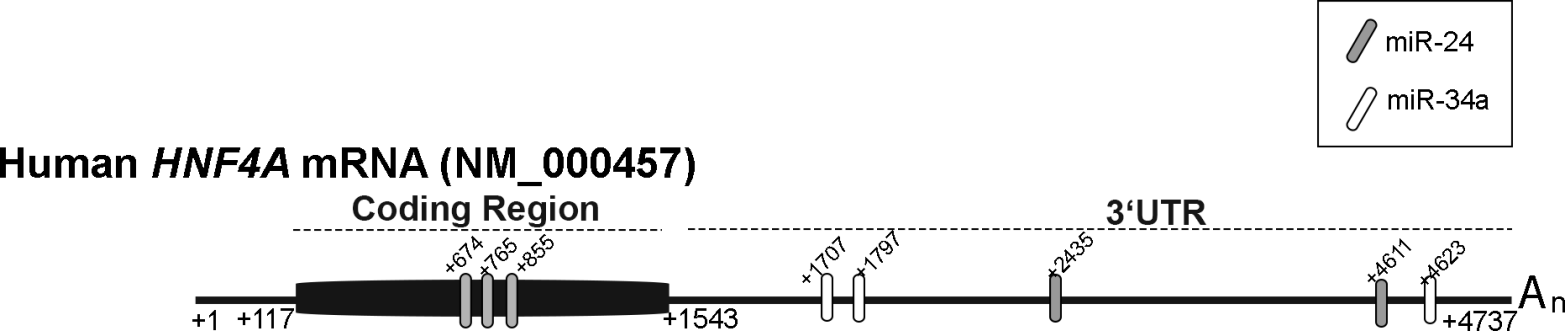
Schematic diagrams of human mRNA of *HNF4A*. [27,28,30]

To study the possible indirect effect of miRNAs on coagulation factor expression, we selected two miRNAs, miR-24 and miR-34a, previously pointed as direct inhibitors of HNF4α [27,30]. Both miRNAs bind to several sites in human *HNF4A*, as described in Fig 3. While miR-34a interacts in three different sites located within *HNF4A* 3’UTR, miR-24 mostly inhibits HNF4α expression by binding to sites located within the coding region [27] (Fig 3).

#### MiR-24 regulates coagulation factors by targeting *HNF4A*

**Fig 4:**
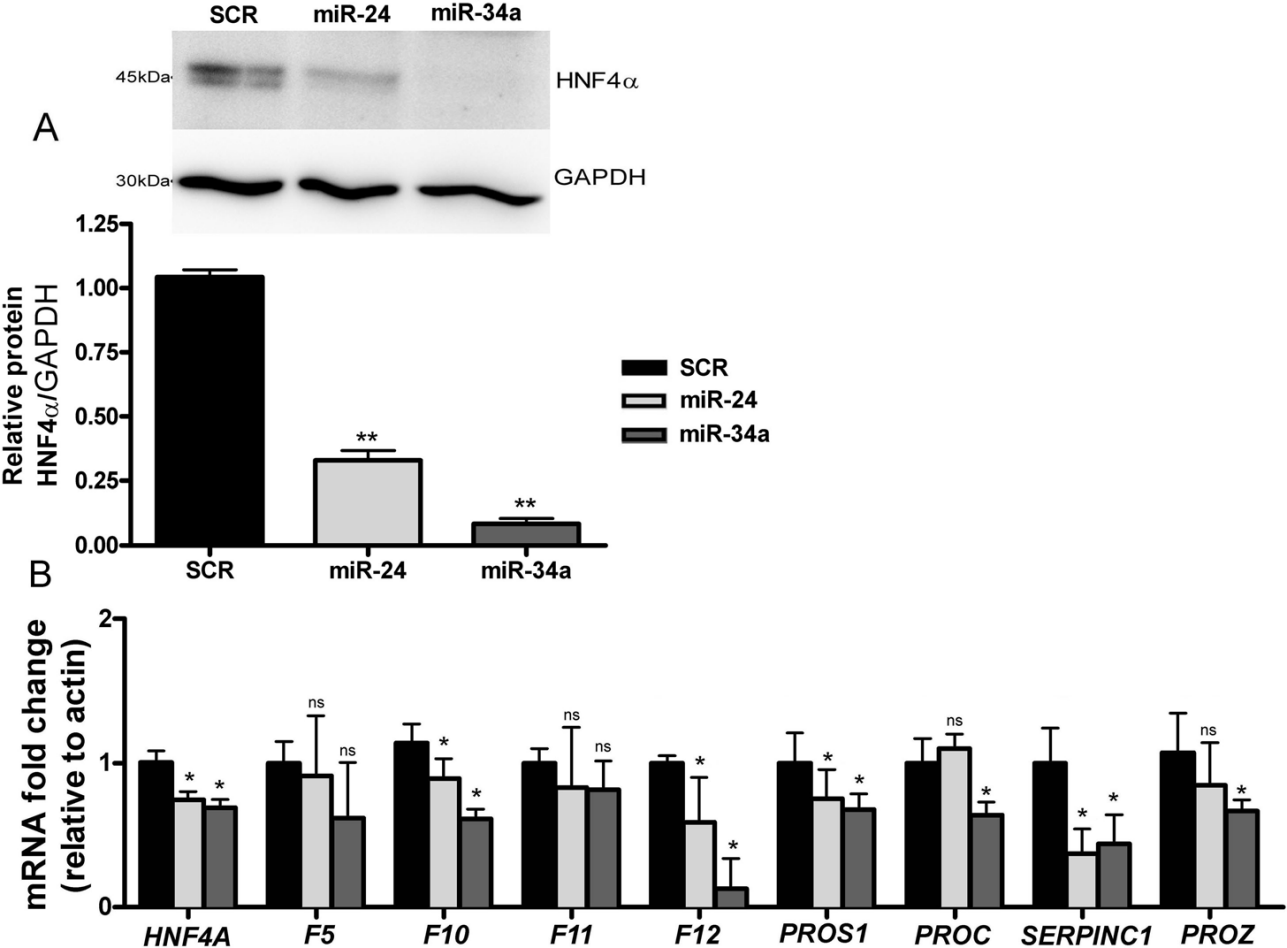
Effect of miRNAs on *HNF4A* and coagulation factors expression in HepG2. HepG2 cells were transfected with 100 nM mimic precursors of miR-24, miR-34a or SCR. Protein lysates and total RNA were obtained after 48 h incubation and analyzed. A) Densitometric analysis of HNF4α protein expression transfected with SCR, miR-24, and miR-34a; representative western blot. B)qRT-PCR analysis of *HNF4A* and coagulation factors mRNA expression. The asterisk indicates a statistically significant difference (P < 0.05) and “ns” indicates a statistically non-significant difference (P > 0.05). Results are mean ± SD of three independent experiments performed in triplicate.

The functional role of miR-24 in regulating HNF4α downstream targets was tested in HepG2 cells by transfecting with miRNA mimics. As expected, results from western blot analysis using whole cell lysates from HepG2 confirmed a decrease of 70% of HNF4α mediated by miR-24 (p = 0.01) (Fig 4A) and a decrease of 25% in mRNA levels (Fig 4B). To investigate whether the decrease of HNF4α was accompanied by a decrease of coagulation factors, we determined mRNA levels by qRT-PCR analysis. HepG2 transfection with miR-24 caused a decrease in mRNA of all selected factors although such reduction was only statistically significant for *F10*, *F12*, *PROS1* and *SERPINC1* (Fig 4C).

#### MiR-34a regulates coagulation factors by targeting *HNF4A*

The functional role of miR-34a on HNF4α was tested in HepG2 cells. Similarly to that seen for miR-24, western blot analysis of lysates from HepG2 showed a significant decrease of HNF4α (Fig 4A) and *HNF4A* mRNA (Fig 4B), as previously described [27,30].

We next investigated the consequences of HNF4α inhibition by miR-34a on coagulation factors. For this, mRNA levels of selected factors were tested by qRT-PCR. As shown in Fig 4B, miR-34a induced a decrease in the expression of all tested factors.

The transcript decrease was significant for *F10*, *F12*, *PROS1*, *PROC*, *SERPINC1* and *PROZ* (Fig 4C).

Overall, these results confirmed the role of miR-24 and miR-34a in regulating *HNF4α* expression and showed a new trans-mechanism of regulation of several coagulation factors by miRNA through HNF4α.

### Inverse correlation between *HNF4A* and both miR-24 and miR-34a expression levels in human livers

**Fig 5:**
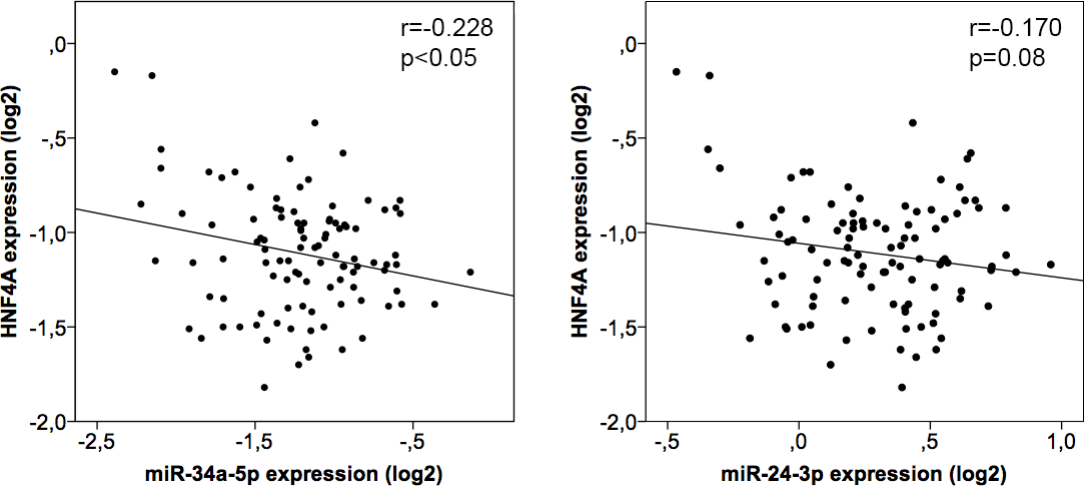
Linear regression analysis between endogenous mature miRNAs (miR-24and miR-34a) levels and *HNF4A* mRNA.qRT-PCR were performed in total RNA purified from healthy livers (n = 104). Statistical significance was taken as p<0.05. The results are presented as Log2 fold change with respect to the normalization standard.

To verify the impact of miR-24 and miR-34a variations on *HNF4A*and because substantial variability in the expression of *HNF4A* and its downstream coagulation genes targets remains unexplained, we analyzed *ex vivo* miRNA-mRNA correlations in human liver samples. We found a slight inverse correlation between *HNF4A* and both miR-24 and miR-34a hepatic transcript levels (r = -0.170; p = 0.08 and r = -0.228; p<0.05; respectively) (Fig 5).

**Fig 6:**
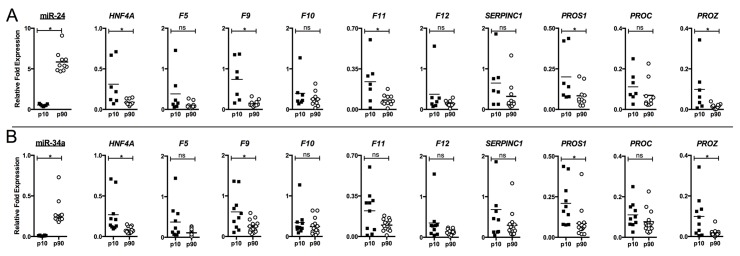
Expression range of miR-24 and miR-34a with *HNF4A* mRNA and its downstream targets in healthy livers. Dot plot diagram of *HNF4A* mRNA, selected coagulation factors and miR-24 (A) or miR-34a (B) levels in livers. P10 and p90 represent 10^th^ and 90^th^ percentiles of miR-24 (A) and miR-34a (B), respectively. Each data point represents an individual liver tissue sample. The results are presented as fold change with respect to the normalization standard. The asterisk indicates a statistically significant difference (P < 0.05) and “ns” indicates a statistically non-significant difference (P > 0.05).

Interestingly, liver samples with extreme miRNA levels (percentiles 10^th^ and 90^th^) showed inverse levels of *HNF4A* and coagulation factors transcripts, in some cases, such correlations were statistically significant (Fig 6). As shown in Fig 6A, liver samples with lower expression of miR-24 had significantly higher expression of *HNF4A*, *F9*, *F11*, *PROS1* and *PROZ*. In turn, liver samples with lower levels of miR-34a also had the following higher coagulation factors levels: *HNF4α*, *F9*, *PROS1* and *PROZ* (Fig 6B).

## Discussion

Previous data from our group showed a noticeable variability in transcript levels of *F11* and *F9* in human healthy livers [33] suggesting that a common genetic regulator is behind specific clustering patterns for human hepatic coagulation factors. However, molecular basis underlying the wide coagulation factors variability in normal population probably involve a combination of factors. Transcriptional factors are ideal candidates to investigate in this framework given their crucial role in controlling mRNAs processing. Moreover, the tandem of some transcription factors and miRNA is an essential network for several biological processes, and its identification has elucidated some of its functions in inflammation [34] and drug metabolism [35] but it still remains to be described in hemostasis. Therefore, we aimed to investigate the contribution of miRNAs as novel participants in the variable expression of coagulation factors as well as to investigate the role of HNF4α in this regulatory loop.

Our first interesting finding was positive correlations between *HNF4A* and the mRNAs of several coagulation genes. To our knowledge these are the first data describing transcriptional relationship between *HNF4A* and a large number of hepatic coagulation factors in human liver.

The bibliographic review ofstudies that experimentally validated *HNF4A* regulation by miRNA, conducted us to select miR-24 and miR-34a as indirect regulators candidates of several coagulation factors [27,30]. Our *in vitro* results showed that miR-24 and miR-34a had a significant impact on the expression of coagulation factors in HepG2 (Fig 4B). Similarly, both miRNAs inhibited HNF4α expression in human hepatocytes, suggesting a TRANS-regulation of several coagulation factors under miRNA control as a novel mechanism in hemostasis.

Additionally, we investigated whether the integrated regulatory association of miR-24, miR-34a, and *HNF4A* observed *in vitro* could also take place in human healthy livers. We first tested the relationship between *HNF4A* mRNA and both miRNA levels confirming that liver samples with higher *HNF4A* transcript are those with the lower miRNAs levels, and *vice versa*. The idea that miR-24 and miR-34a gene regulation is mediating as a significant mechanism contributing to variation in gene expression has been previously documented. Lamba *et al* [35] demonstrated that miR-34a expression was significantly and negatively correlated with expression levels of multiple hepatic transcription factors (including HNF4α) and that it was involved in a significant mediation of the association observed between CYP2C19 and HNF4α [35]. Then, our results while confirming the miR-34a/*HNF4A* interaction, for the first time also showed a key regulatory role of miR-34a in hepatic coagulation genes in humans.

In turn, we also confirmed here a regulatory connection between miR-24 and *HNF4A*, as Hatziapostolou *et al* did in samples from 12 healthy livers [34]. Our series of human liver samples extended to 104, and provide additional consequences for miR-24/*HNF4A* interaction, as it had repercussions on the levels of coagulation factors transcripts. Of note, liver samples with extreme expression of miR-24 levels (percentiles 10^th^ and 90^th^) were, in the opposite, those with utmost *HNF4A* and coagulation factors expression. Similar findings can be inferred from data reported by Hatziapostolou *et al* [34].Thus, these authors described that transient inhibition of *HNF4A* in an hepatocellular carcinoma model drives a feedback loop circuit through several inflammatory miRNAs, and among them miR-24 [34]. Moreover, our *in vitro* data supported a lower inhibitory effect for miR-24 in comparison with miR-34a, which might explain that only statistically significant values are found for liver samples from extreme percentiles (Fig 6). Alternatively, the sample size might be insufficient to reach statistical power (Fig 5).

In summary, our results suggest that miR-24 and miR-34a participate in the interindividual variability observed in expression of coagulation factors genes in humans. Therefore, in both circuits *HNF4A* would work as a key switchboard. It remains to be clarified to what extent genetic variations of miRNAs that change HNF4α expression and activity may affect the expression of hepatic coagulation factors, and to what extent this may affect the development of thrombotic or hemorrhagic disorders.

## Supporting Information

S1 Table TaqMan® probes used for real time quantitative PCR of human genes. (DOCX)
