# Supplementary material for: MiRNA-Based Regulation of Hemostatic Factors through Hepatic Nuclear Factor-4 Alpha
Source: PLoS One. 2016 May 2;11(5):e0154751. doi: 10.1371/journal.pone.0154751 (PMC4852917; doi:10.1371/journal.pone.0154751)
Supplement: S1 Table — (DOCX) [file pone.0154751.s001.docx]

**S1Table**: TaqMan® probes used for real time quantitative PCR of human genes

| *Human symbol Gene* | TaqMan® probe |
| --- | --- |
| *F5* | Hs00914120_m1 |
| *F8* | Hs00252034_m1 |
| *F10* | Hs00984443_m1 |
| *F12* | Hs00166821_m1 |
| *SERPINC1* | Hs00166654_m1 |
| *PROC* | Hs00165584_m1 |
| *PROZ* | Hs00187370_m1 |
| *PROS1* | Hs00165590_m1 |
| *ACTB* | Hs99999903_m1 |
| *HNF4A* | Hs00230853_m1 |
